# Inhibitory Effect of *Viola odorata* Extract on Tumor Growth and Metastasis in 4T1 Breast Cancer Model

**Published:** 2018

**Authors:** Hiva Alipanah, Mohammad Reza Bigdeli, Mohammad Ali Esmaeili

**Affiliations:** a *Faculty of life sciences and biotechnology, Shahid Beheshti University, G.C., Tehran, Iran. *; b *Department of Biology, Medicinal Plants and Drugs Research Institute, Shahid Beheshti University, Tehran, Iran.*

**Keywords:** *Viola odorata*, Antioxidant, Metastasis, Apoptosis, Cancer

## Abstract

*Viola odorata* as a medical herb is used in liver disorders and relieving cancer pain. In the present study, the cytotoxic, antioxidant, and anti-metastatic properties of *Viola odorata *hydro-alcoholic extract (VOE) were investigated in 4T1 breast cancer model. After treatment of 4T1 breast cancer cells with VOE, cell viability was measured by MTT assay. The implanted mice were treated with different concentration of VOE (50, 150 and 250 mg/kg) for 21 days. Levels of lactate dehydrogenase (LDH), γ -glutamyl transferase (GGT), alkaline phosphatase (ALP), carcinoembryonic antigen (CEA) and cancer antigen 15-3(CA15-3) in serum, and also catalase (CAT) and superoxide dismutase (SOD) activities in tumor tissue were measured. Metastatic rate was investigated in liver, spleen and lung tissues. VOE decreased cell viability of 4T1 cells, significantly. VOE significantly inhibited the cell proliferation, but not vasculature in the tumors that revealed by immunohistochemical analysis for Ki-67 and CD31 expression, respectively. VOE increased the Bax/Bcl-2 ratio in VOE250-treated group compared to control group. Serum analysis showed that treatment with 250 mg/kg of VOE significantly reduced LDH (not ALP and GGT) levels compared to controls. No linear correlation was found between the values of CEA and CA15-3 with tumor size. The rate of CAT activity was increased in VOE250-treated rats whereas, CAT and SOD activities were reduced in VOE50 group. VOE250 significantly decreased the metastatic rate in liver and lung compared to the other doses of VOE. Consequently*, Viola odorata *has cytotoxic effects on 4T1 cells and affects antioxidant activity and metastasis in breast cancer.

## Introduction

Breast cancer intrinsically is a systemic and the most common malignant disease in women and starts as a local disease and spreads (metastasize) to the lymph nodes and distant organs, such as the lungs, liver, bones and brain (1). Although, metastasis in breast cancer patients is the main cause of death, the rate of metastasis and mortality can be decreased by early diagnosis via various methods, such as mammographic screening (1). Nevertheless, novel and more effective therapeutic options for breast cancer in advanced stage are still needed.

*Viola odorata *as a member of the *Violaceae* plant family is commonly used in Iranian traditional medicine (2). In the traditional system, *V. odorata *has been characterized as a medical herb which is widely used in anxiety, lower blood pressure, bronchitis, kidney and liver disorders and also relieving cancer pain (3, 4). A number of reports have also indicated anti-inflammatory, antipyretic, antioxidant and antibacterial activities of *V. odorata *(2, 5, 6). Fresh leaves of *V. odorata* have been used internally and externally in the treatment of cancer. Decoction, poultice, infusion of leaves, syrup made from petals or a liquid extract of *V. odorata* fresh leaves are used for throat and tongue cancers (4, 7-9). Additionally, some *Violaceae* compounds (cyclotide and flavonoids) demonstrated significant anti-carcinogenic and antioxidant activity(10, 11). 

The aims of this study were to determine: 1) whether *V. odorata* extract would effectively decrease the size of tumors in 4T1 breast cancer mouse model, 2) whether there would be changes in metastatic rate, expression of Ki-67 (cell proliferation) and CD31 (angiogenesis) determined by histopathological studies in mice treated with *V. odorata*, 3) the changes in antioxidant enzyme activity (SOD and CAT), liver enzymes (LDH, ALP, GGT) and tumor markers (CA15-3 and CEA) levels in response to *V. odorata*. To the researcher’s best knowledge, this is the first study of this kind to assess the therapeutic effectiveness of *V. odorata* in the 4T1 breast cancer model.

## Experimental

*Ethics statement*

The experiments were performed based on the Principles of Laboratory Animal Care by the US National Institute of Health (NIH publication No. 85-23, revised 1985).

*Cell culture*

4T1 mouse mammary carcinoma cells were purchased from the Pasteur Institute of Iran (C604) and cultured in 75 cm^2^ culture flasks with Dulbecco’s modified Eagle’s medium (DMEM) supplemented with 10% fetal bovine serum, penicillin (100 IU/mL) and streptomycin (100 mg/mL) until they were 80–90% confluent.

*MTT assay*

Cells were seeded at a density of 1×10^4^ cells/mL in 96-well polystyrene culture plates at 37 °C with 5% (v/v) CO_2_ for 24 h. After incubation, cells were incubated with various concentrations of VOE (5, 10, 30, 40, 50, 60, 80,100,150 and 200 µg/mL). Control cells received only culture medium. Then, 100 µl of MTT solution (5 mg/mL) was added to each well and the plates were incubated at 37C for an additional 3 h. After dissolving the formazan crystals, cells were incubated with detergent at 37 °C. Absorbance was measured in each well, including the blanks at 570 nm by ELISA Plate Reader. Cell viability (%) was calculated for all groups compared to control sample. All experimental samples were performed in triplicate.

*Viola odorata Hydro-alcoholic Extract*

Aerial parts of *V. odorata *were collected in summer from Rostamabad, Gilan, Iran and authenticated by Dr. Mohammad Reza Kanani. The plants were kept in the herbarium (Index Herbarium code: MPH-615) for future reference. Water-ethanol extract is a specific solvent for extracting antioxidants and polyphenolic compounds like flavonoids. In this study, the traditional solvent extraction (TSE) (50:50) method was used (12). Department of Biology, Medicinal Plants and Drugs Research Institute (Shahid Beheshti University, Iran) prepared hydro-alcoholic (50:50) extract.

*DPPH radical scavenging activity (DPPH assay)*

The scavenger activity of VOE was investigated by DPPH *(*α, α-diphenyl-β-picrylhydrazyl) assay. The solution of *V. odorata *(2 mg/mL) was prepared in methanol. The concentration of DPPH solution was 0.08 mg/mL in methanol. Synthetic antioxidant, Butylated hydroxytoluene (BHT) in 0.5 mg/mL concentration was used as a reference. VOE with different concentrations (2, 5, 10, 20, and 30 mg*/*mL) was transferred by pipette into columns of a 96-well micro plate. For methanol evaporating, plate was left at room temperature. In the first column, methanol (187 μl) and DPPH (63 μL) were added to four wells (control column) and 250 μL of methanol was added to remaining wells. The plate was shaken and allowed to stand in the dark place at room temperature for 70 min. Absorbance was measured in each well at 517 nm using plate reader. The following formula was used to calculate inhibition percentage (Percentage of free radical scavenging activity):

$\mathrm{\%Af}=\frac{\mathrm{Ac}-\mathrm{As}}{\mathrm{Ac}}\times 100$

*Ac *is control absorbance, *As *is sample absorbance and *Af *is final absorbance. IC_50_ values are mean*±*SEM, n = 4. The lower IC_50_ in DPPH indicated a higher antioxidant activity.

*HPLC of Viola odorata hydro-alcoholic extract*

Different phytochemicals were isolated from all extracts i.e. n-hexane, butanol, methanol and aqueous extracts of *V. odorata *like glycoside, saponins, methyl, salicylate, cyclotides, flavonoids, alkaloids, triterpenoids, mucilage and vitamin C (7, 13-15). *In-vivo* and *in-vitro* studies show that flavonoids have protective effects against many types of cancers(16). Therefore, HPLC fingerprint was applied for qualification of the *Viola odorata *extract and detecting the presence of flavonoids. HPLC protocol was used as described by Siddiqui  et al. 2011 (17). *V. odorata *was extracted and dissolved in methanol at room temperature for 24 h. 10μL application volume was injected into HPLC and peaks with same retention time (RT) values with the standards were recorded.

*Animal model*

Tumor transplantation was carried out according to the modified method of *Pulaski et al,* (2001) (18). Female BALB/c mice (n = 20) weighing 18-20g at the age of 7-8 week were purchased from the Pasteur Institute, Karaj, Iran. 4T1 cells, cultured *in-vitro*, were harvested and re-suspended serum free media. The animals received 0.1 mL subcutaneous injections of cell suspension (0.8 million cells) in the right mammary gland or hind flank. The animals were kept in animal house in standard condition and feed with standard pellet diet and water ad libitum, under controlled temperature (22 ± 0.5 °C) and light (12 h light/ dark cycle).

*Experimental Protocol*

Animals were randomly divided into four groups (n=5). The first (VOE250), second (VOE150) and third (VOE50) groups were treated with 250, 150, and 50 mg/kg/day of the VOE for 3 weeks, respectively. The fourth group as the control group (Cont), received distilled water by gastric gavage. The mice were sacrificed on 22^th^ day. The following formula was used to calculate the tumor inhibition rate and tumor volume:

Tumor inhibitory rate = (tumor weight of the control group − tumor weight of experimental group)/tumor weight of the control group ×100.

Tumor volume = ½ (length × width^2^)

*Blood biochemical assays*

The blood samples were obtained from the heart of animals. In addition, 6 normal mice were used for obtaining baseline levels, on 22^th^ day. The serum samples were collected after blood centrifugation at 3000 rpm for 10 min. These samples were used for determination of liver enzymes (GGT, ALP and LDH) and tumor markers (CEA and CA15-3) levels by means of commercial kits. 

*Determination of catalase and superoxide dismutase activities*

CAT and SOD enzymes activities were assessed by the following method of *Genet et al*., (2002) (19) with some modifications. Catalase buffer was made by adding 0.05 mL H2O2 to 50 mL of sodium phosphate buffer and the rate of H_2_O_2 _decomposition was followed by monitoring absorption at 240 nm for 2 min. Superoxide dismutase buffer was made by adding 0.0018 gr EDTA and 0.003 gr pyrogallol to 50 mL of sodium phosphate buffer. The absorbance change at 420 nm of the mixture was monitored for 3 min. 

*Immunohistochemical Analysis of Ki-67 and CD31 and histopathological studies*

Liver, lung and spleen tissues were fixed, dehydrated and embedded in paraffin. Sections were stained with hematoxylin and eosin (H&E). Histopathological analysis was used based on H&E staining to identify tumor metastatic and necrotic area by ImageJ software as a percentage of total area. Tumor sections of control and VOE-treated mice were processed for immunohistochemical analysis of Ki-67 (Dako) expression to assess cell proliferation, and CD31expression (Dako) to 

visualize blood vessels.

*Western Blotting*

The expression of Bax and Bcl-2 proteins were evaluated in the experimental groups (VOE250, 150, 50) and control group by Western blotting following sample extraction and SDS-PAGE. Breast cancer tissue samples were lysed in RIPA buffer (150 mM NaCl, 1% NP-40, 50 mM Tris PH=8.0, 1% SDS, 0.5% sodium deoxycholate, 1mM EDTA and protease inhibitor cocktail) and centrifuged at 12,000 rpm at 4 ºC for 20 min. 

SDS sample buffer was added to aliquots of tissue extracts. Samples were heated at 100 ºC for 5 min. Protein was separated by 10% SDS-PAGE. Blots were incubated with specific primary polyclonal rabbit antibodies against Bax (1:500 dilution) (Santa Cruz), Bcl-2 (1:500 dilution) (Santa Cruz) and β-actin (1:1000 dilution) (Santa Cruz) in TBS-T for 18h. Then, they were incubated by secondary anti-rabbit (1:500 dilution) (Santa Cruz) in TBS-T for 90 min separately. Bax and Bcl-2 immune-reactive proteins were detected with advanced chemiluminescence (Enhanced Chemiluminescence, Amersham Biosciences) and exposed to a film. The signal intensity of the blots was measured by an image analysis system (Image j, version 1.46r).

*Statistical Analysis*

The obtained results were expressed as mean ± SD. Comparison with appropriate control was performed using one-way ANOVA. Correlation levels between tumor markers with tumor size and liver enzyme with metastatic rate were analyzed by a Pearson correlation coefficient (R). The least significant difference (LSD) was used as the post-hoc test. *P *< 0.05 was considered significant. Data was analyzed using the statistical package “SPSS 16.0”.

## Results

*DPPH assay*

**Figure 1:**
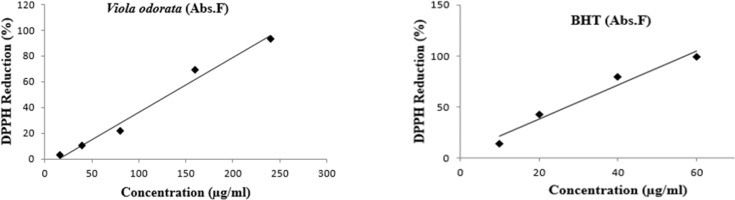
DPPH radical scavenging activity (%) of hydro-alcoholic extract of *Viola odorata*. BHT is standard in different concentration. VOE: *Viola odorata *extract, (n = 4).

The radical scavenging activity of VOE was evaluated using DPPH assay. VOE showed high total antioxidant activity with a low IC_50_ (131.97 μg/mL). The free radical scavenging activity of hydro-alcoholic extract of *V. odorata *showed a dose dependent increase in the percentage antioxidant activity (Figure 1). Percentage inhibition of the extract at the concentration of 16 μg/mL was 3.01 ± 0.24 and it was 93.07 ± 0.28 for 240 μg/mL. The free radical scavenging activity of BTH was measured as well, and the IC_50_ value was 27.30 μg/mL.

*HPLC of Viola odorata hydro-alcoholic extract*

In the present study, HPLC was used for flavonoids run in methanol. HPLC profile of VOE showed presence of several flavonoids and peaks at retention time 1.97 (luteolin), 2.06 (quercetin), 2.50 (apigenin), 2.73 (kaempferol) 3.352, 3.910, 5.706, 8.59, 11.243, 13.37. The results showed that different compounds in *V. odorata *extract (Table 1).

*Cell viability*

**Figure 2:**
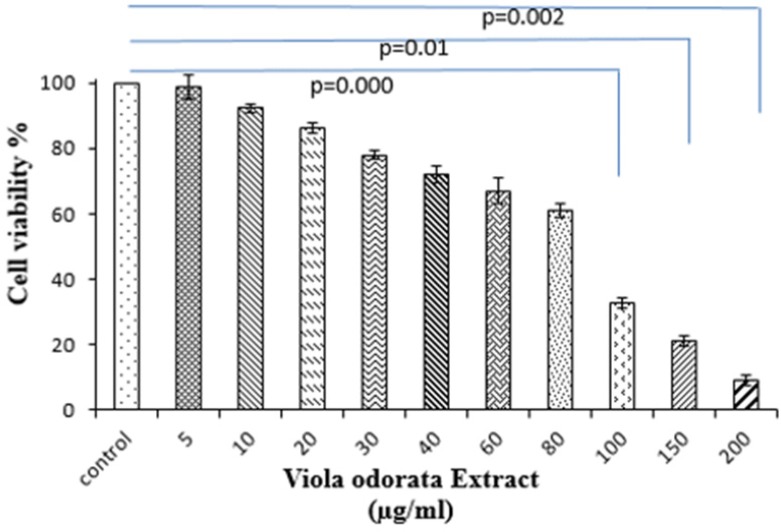
*Viola odorata* extract reduces the viability of 4T1 cells. Cells were seeded at a density of 1×10^4^ cells/mL in 96-well polystyrene culture plates at 37 °C with 5% (v/v) CO_2_ for one day. After 24 h of incubation, cells were incubated with *Viola odorata* extract at the indicated concentrations for 24 h and then processed and assayed using MTT assay kit. Each value represents the mean ± SD of three experiments. (n = 3).

We performed the MTT assay with different concentrations of VOE to assess whether VOE acts on the *in-vitro* viability of 4T1 cells. As shown in Figure 2, treatment with 5 µg/mL to 80 µg/mL of VOE did not affect the cell viability after 24 h (*P *> 0.05) while, concentration of 100, 150 and 200 µg/mL of VOE significantly decreased the cell viability. Based on these results, the 4T1 cell is highly sensitive to hydro-alcoholic extract of* V. odorata.*

*Body weight*

**Figure 3:**
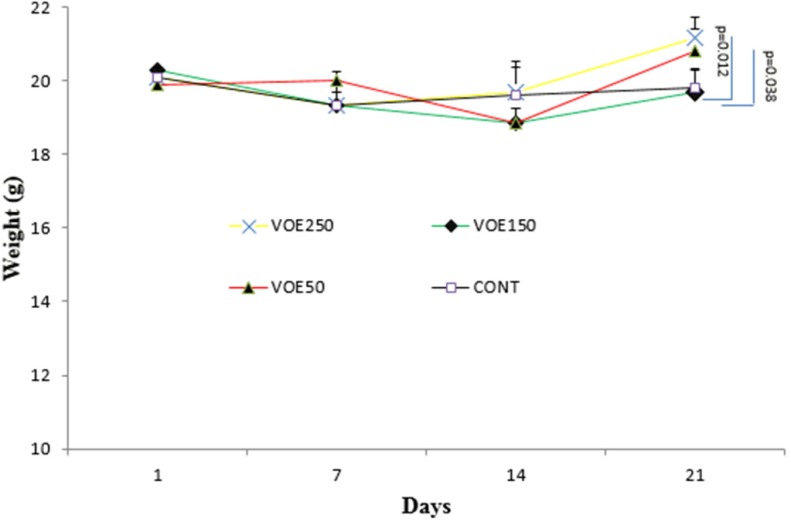
Body weight in BALB/c mice treated with *Viola odorata *extract. Body weight was measured at days 1,7,14 and 21. VOE 250, 150, 50: *Viola odorata* extract in different concentration (250, 150 and 50 mg/kg b w), Cont: control group, (n = 5

Body weight of the animals was measured on 1^st^, 7^th^, 14^th^ and 21^st^ days. The Body weight in mice treated with *V. odorata *extract (VOE250 and VOE50) was significantly higher than the control group on 21^st^. There was no statistically significant difference in body weight between VOE150 and the control group (Figure 3).

*Tumor Size*

**Figure 4:**
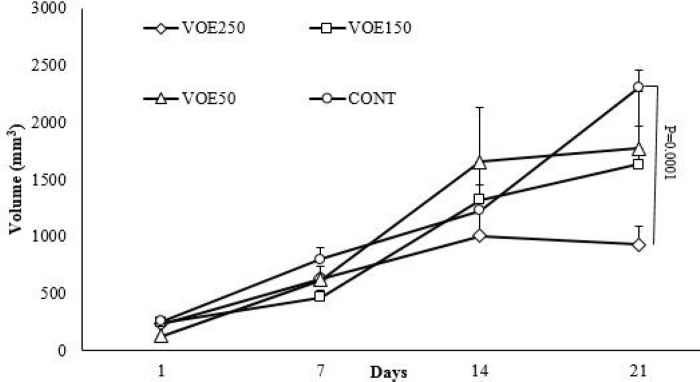
Effect of *Viola odorata* extract on serum enzymes in normal and treated group (A: ALP and B: LDH level in the serum of treated, control and baseline group). VOE250, 150, 50: *Viola odorata* extract in different concentration (250, 150 and 50 mg/kg b.w), Cont: control group, ALP: Alkaline phosphatase, LDH: Lactate dehydrogenase, (n = 5).

Mean tumor volumes of mice treated with *V. odorata* are shown in table 2 and Figure 4. The size of tumors in the extract fed mice (VOE250) was smaller than in water fed mice on 21^st^, but the mean tumor volume of the VOE150 and VOE50 groups was not significantly different compared to the control group. We confirmed that continuous feeding of *V. odorata* (250 mg/kg) significantly inhibited the growth of cell-derived tumors by 42.56 % compared to the untreated control group (Table 2).

*LDH, ALP and GGT Levels*

**Figure 5:**
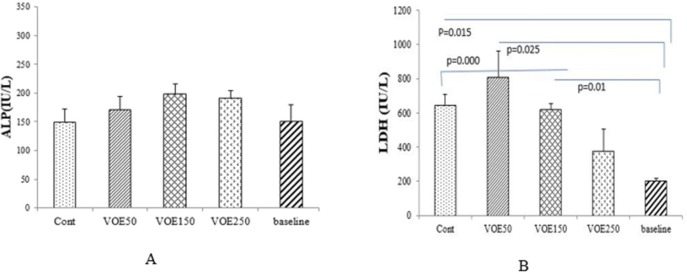
Effect of *Viola odorata* extract on CEA level. A: CEA in the serum of treated and control group. B: Correlations between CEA level and tumor size. VOE 250, 150, 50: *Viola odorata* extract in different concentration (250, 150 and 50 mg/kg b.w), Cont: control group, (R): Pearson correlation coefficient, (n = 5

The effects of the VOE on some serum enzyme levels are shown in Figure 5. There were significant differences (*P* < 0.05) in the serum LDH of the cancer groups (Cont, VOE50 and VOE150) compared to base line level. LDH level in VOE250 was lower than the control group. ALP and GGT enzymes showed no statistical increase in VOE-treated groups compared to the control group (Figure 5). Data related to changes in GGT has not been shown.

*CEA and CA15-3 (tumor markers) Levels*

**Figure 6:**
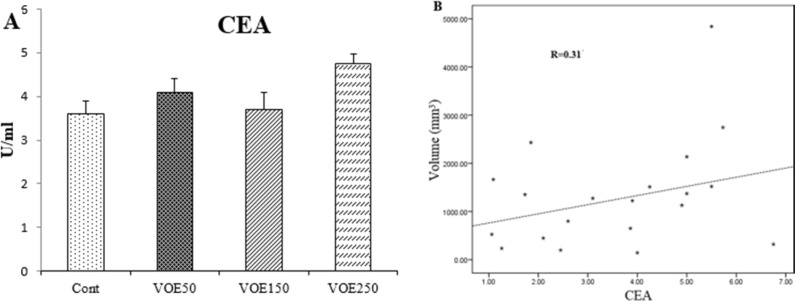
Effect of *Viola odorata* extract on CA15-3 level. A: CA15-3 in the serum of treated and control group. B: Correlations between CA15-3 level and tumor size. VOE 250, 150, 50: *Viola odorata* extract in different concentration (250, 150 and 50 mg/kg b.w), Cont: control group, (R): Pearson correlation coefficient, (n =5).

**Figure 7:**
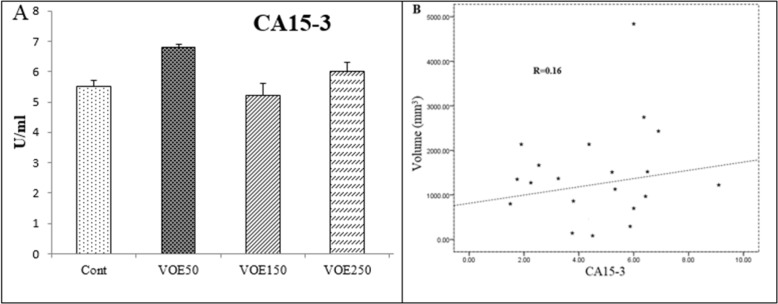
Effect of *Viola odorata* extract and on level of CAT activity in 4T1 breast cancer mouse model. VOE 250: *Viola odorata* extract, VOE 250, 150, 50: *Viola odorata* extract in different concentration (250, 150 and 50 mg/kg b.w), Cont: control group, (n = 5). CAT: catalase, Data are shown as (mean ± S.D) per100

In this study, CEA and CA15-3 markers showed no statistical change in cancer groups compared to the control group (Figure 6, 7). No linear correlation was found between the values of CEA (R = 0.27) (Figure 6B) and CA15-3 (R = 0.19) (Figure 7B) with tumor size. CEA and CA15-3 levels in experimental groups were not significantly changed in comparison to control.

*CAT and SOD Activity*

**Figure 8.**
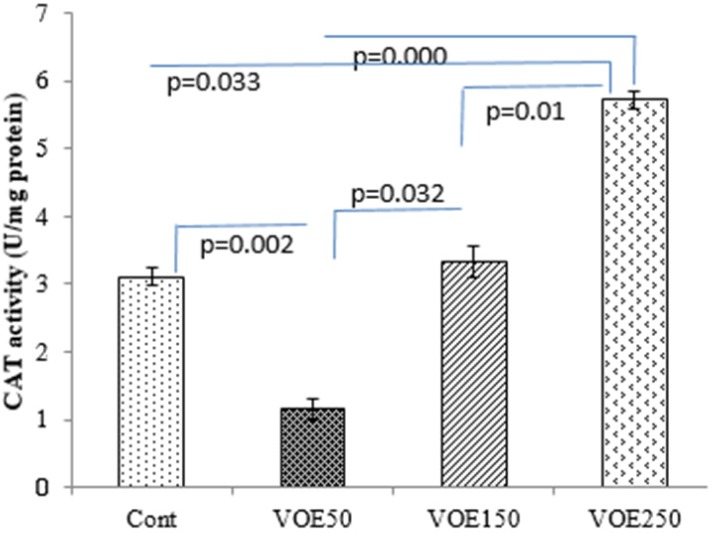
Effect of *Viola odorata* extract on level of SOD activity in 4T1 breast cancer mouse model. VOE 250: *Viola odorata* extract; VOE 250, 150, 50: *Viola odorata* extract in different concentration (250, 150 and 50 mg/kg b.w), Cont: control group, (n = 5). SOD: superoxide dismutase: Data are shown as (mean ± S.D) per100

**Figure 9:**
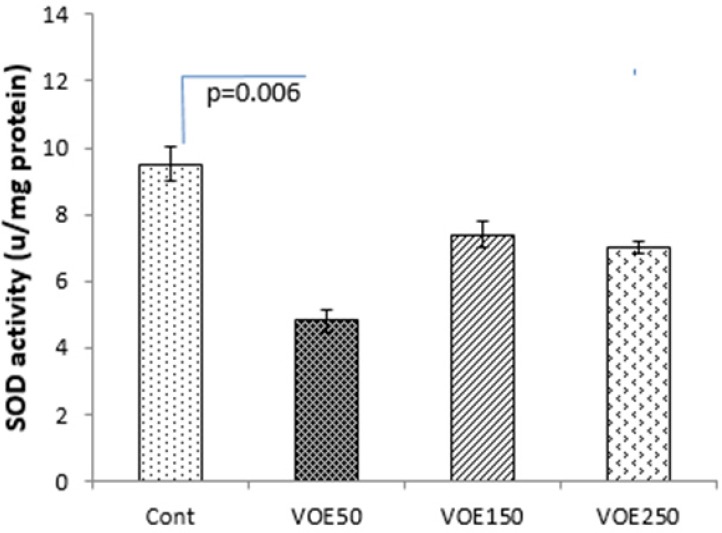
The levels of tissue metastasis (%) cells in 4T1 breast cancer mouse model. VOE 250, 150, 50: *Viola odorata* extract in different concentration (250, 150 and 50 mg/kg b.w), Cont: control group, (n = 5

Treatment with 250 mg/kg of *V. odorata *extract (VOE250) increased the CAT activity (*P *< 0.05) compared to the control group, significantly. The results showed that CAT and SOD activities were decreased in the VOE50 group in comparison with the control group (Figure 8, 9).

*Histopathological studies (Tumor Necrosis and Tissue Metastasis)*

**Figure 10:**
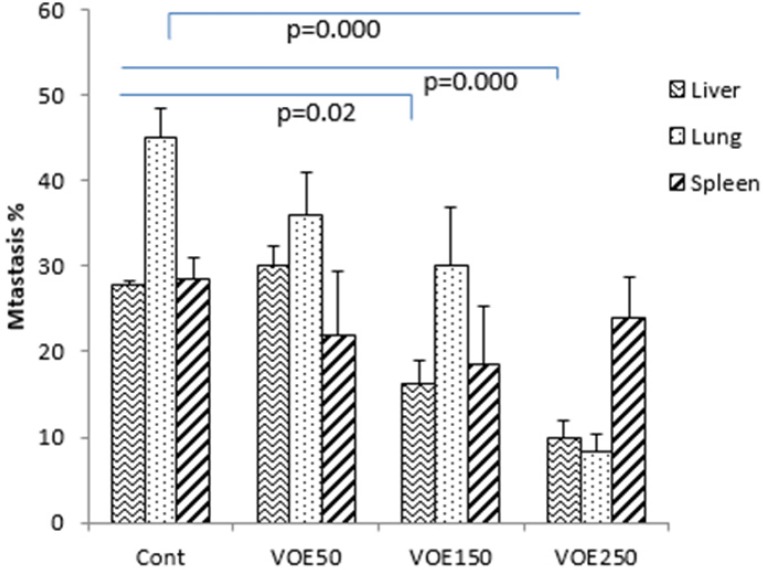
The levels of tumor necrosis (%) in 4T1 breast cancer mouse model. VOE 250, 150, 50: *Viola odorata* extract in different concentration (250, 150 and 50 mg/kg b.w), Cont: control group, (n = 5

**Figure 11:**
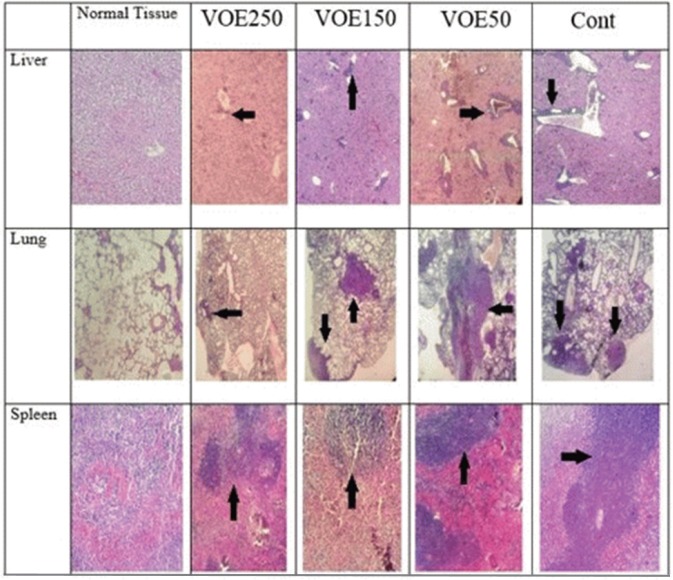
4T1 mouse mammary tumor metastasis in histological study. VOE 250: *Viola odorata* extract (250 mg/kg b.w.) group; VOE 250, 150, 50: *Viola odorata* extract in different concentration (250, 150 and 50 mg/kg b.w), Cont: control group, (n = 5

**Figure 12:**
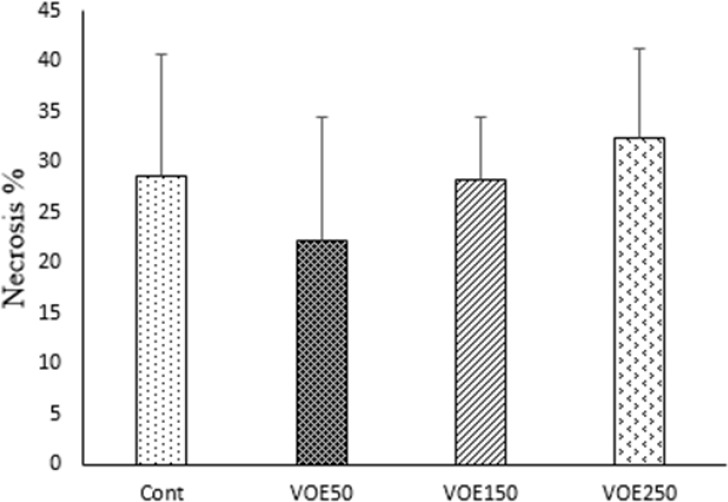
The levels of tumor necrosis (%) in 4T1 breast cancer mouse model. VOE250, 150, 50: *Viola odorata *extract in different concentration (250, 150 and 50 mg/kg b.w), Cont: control group, (n = 5).

The liver metastatic area was found very close to vessels. As it’s observed in Figure 10 and 11, the rate of liver metastasis in groups received *V. odorata* extract (150, 250 mg/kg) was notably decreased in comparison with the control group. On the other hand, the most decreasing rate in the level of lung metastasis was diagnosed in histopathological slides of VOE250 group, which received *V. odorata *(250 mg/kg) (Figure 11). Spleen metastasis showed no statistical decrease in the treated groups compared to the control group. The rate of tumor necrosis in mice which received *V. odorata* extract (VOE250, VOE150, and VOE50) was not different compared to the control group (Figure 12).

*Immunohistochemical Analysis of cell proliferation and neovascularization*

**Figure 13:**
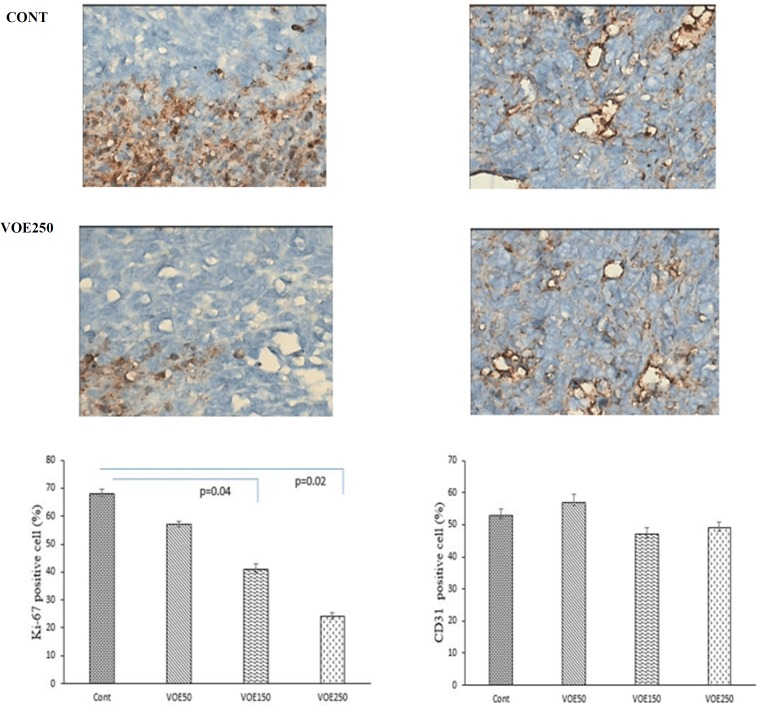
Effect of *Viola odorata *extract on cell proliferation and neovascularization by using tumor tissues from 4T1 breast cancer model

We investigated the effects of VOE on cell proliferation and neovascularization using tumor tissues from 4T1 breast cancer model. Figure 13 shows immunohistochemical analysis for Ki-67 (a cellular marker for proliferation) in the control and experimental groups. VOE reduced Ki-67 expression in the experimental groups compared to the control group. 

The number of Ki-67 positive cells in VOE50, 150 and 250 was lower by about 16%, 39% (*P *<0.04) and 64% (*P *< 0.02) compared to the control group (Figure 13). Figure 13 also shows immunohistochemical analysis for CD31 (angiogenic marker) in the control and treated groups. There was no significant difference in the vessels area (the number of CD31 positive cells) between the experimental and control groups (Figure 13).

*Western blotting*

Bcl-2 family proteins like B-cell lymphoma-2 (Bcl-2) and Bcl-2 associated X protein (Bax) act as anti-apoptotic and pro-apoptotic factors, respectively (20). Increasing the ratio of Bax/Bcl-2 increases apoptosis via the mitochondrial (intrinsic) pathway (20). In this study, the Bax/Bcl-2 ratio was measured as an indicator for apoptosis. Our results indicated that VOE250 increased the Bax/Bcl-2 ratio by downregulating Bcl-2 and upregulating Bax protein expression in 4T1 cells. 

**Figure 14:**
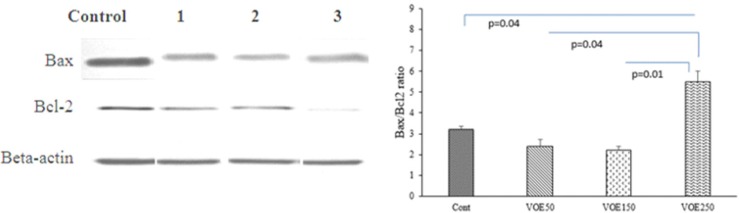
Effect of *Viola odorata *extract on Bax/Bcl-2 ratio. VOE 250: *Viola odorata* extract (250 mg/kg b.w.) group; VOE 250, 150, 50: *Viola odorata* extract in different concentration (250, 150 and 50 mg/kg b.w), Cont: control group, (n = 5

The Bax/Bcl-2 ratio in VOE50 and VOE150 groups was not significantly different in compared with the control group (Figure 14).

## Discussion

In the present study the cytotoxic, antioxidant, anti-angiogenic, anti-metastatic, and apoptotic properties of VOE were investigated in 4T1 breast cancer mouse model. Our results indicated that VOE decreased cell proliferation of 4T1 cell line *in-vitro*. Reduction of tumor growth and metastatic rate and induction of apoptosis were also observed *in-vivo*. Furthermore, VOE increased CAT activity and attenuated LDH and SOD activities. In contrast, VOE showed no significant effect on ALP, GGT, tumor markers levels (CEA and CA15-3), and angiogenesis (CD31). 

**Table 1:** Showing HPLC retention time of flavonoids in *Viola odorata *extract. R_t_: Retention time_, _VOE: *Viola odorata* extract

**Violaodorata**	**R**_t_	**Flavonoids**
136.01	2.05	*quercetin*
Not detected	1.75	*myricetin*
Not detected	2.72	*isorhamntin*
74.82	2.6	*kaempferol*
180.74	1.98	*luteolin*
32.11	2.48	*apigenin*
Not detected	3.75	*rhamentin*

**Table 2:** Effect of *Viola odorata *extract on tumor inhibition rate in breast cancer model. VOE 250, 150, 50: *Viola odorata* extract in different concentration (250, 150 and 50 mg/kg b.w), Cont: control group, (n = 5). * *P*<0.05 compare to control group

**Group**	**Tumor weight (g)**	**Inhibition rate (%)**
CONT	2.96	-
VOE50	2.93	1.01
VOE150	2.05	30.74
VOE250	1.70	42.56^*^

MTT assay revealed that VOE exhibited a dose-dependent cytotoxic effect (IC_50: _95.28 µg/mL) against the proliferation of 4T1 cell line. Previous studies have shown that the cytotoxic effects of *V. odorata* on various cancer cell lines are induced via isolating cyclopeptides (varv A, varv F, and cycloviolacin O2) (21, 22). The other experimental study demonstrated that other species of Viola (*Viola tricolor*) suppressed the proliferation of MCF-7 and Neuro2a cell lines (23). There is abundant evidence indicating flavonoids inhibit cancer cell proliferation(24, 25). The VOE used in this study contains several flavonoids, including luteolin, kaempferol, apigenin, and quercetin. Therefore, this hypothesis reinforce flavonoids and cyclotides contents of *V. odorata *may be responsible for its antitumor effect. 

In our study, the antioxidant capacity of hydro-alcoholic extract of *V. odorata* was confirmed by DPPH assay (26). VOE showed high total antioxidant activity with a low IC_50_ (131.97 μg/mL). Antioxidant capacity of *V. odorata* has been reported in both water and methanol extracts by* Stojković et al.* and *Ebrahimzadeh et al., *respectively (5, 6) while, our results determined that hydro-alcoholic extract of *V. odorata* has more appropriate antioxidant activity than its water (IC_50_:140.7 μg/mL or 163.6 μg/mL) and methanol extract (IC_50_: 245.1 μg/mLl) (5, 6). Therefore, it suggests that higher antioxidant activity of VOE may be related to solvent type and extraction method. Moreover, based on antioxidant property of *V. odorata* the effect of VOE on antioxidant enzyme activity (CAT and SOD) was assessed. It has been reported that natural antioxidants may reduce the incidence of free radical-mediated diseases by increasing of endogenous antioxidant enzymes levels (27). Noticeably, elevation of SOD activity suppresses tumor growth in breast and prostate cancers (28), while the reduction of CAT activity triggers the intracellular hydrogen peroxide production, DNA damage, and progressing of tumor growth (29). In present investigation, there was an increase in the CAT activity in the animals receiving VOE250. It seems that VOE enhances the activity of CAT to protect cells from oxidative injury. In biological system, the most oxidant agents and reactive oxygen species (ROS) affect NF-κB signaling pathway (30, 31). The importance of ROS on NF-κB activation is further supported by various investigations that have demonstrated inhibition of NF-κB activity by antioxidants, such as polyphenols and vitamin E (32). It has also been shown that overexpression of SOD increases NF-κB activation, whereas up-regulation of CAT decreases the NF-κB activation (32-34). NF-κB regulates cell proliferation, angiogenesis, and metastasis of the tumor. (32). Therefore, it can be suggested that increasing of CAT activity and involvement of NF-κB are likely mechanisms for the decrease in tumor growth rate observed in VOE250 group. Our funding also showed a significant decrease in SOD activity in the VOE50 group compared to control. A possible explanation for this unexpected finding is that VOE has an inhibitory effect on SOD activity in lower dose. More investigations to clarify these effects can be the subject for future studies.

Our finding expressed that treatment with VOE exerted a considerable decrease in tumor growth rate and volume. Unlike the control and VOE50 groups, none of mice treated with VOE150 and VOE250 showed any lethargy during the treatment. Our immunohistochemical examinations also indicated that VOE reduced cell proliferation (ki-67) while, had no effect on angiogenesis (CD31). The inhibition of tumor growth by VOE250, as evidenced by a decrease in tumor volume and cell proliferation (ki-67), may be attributed to the inhibition of cell survival signaling in 4T1 cells. In fact, inhibition of tumor angiogenesis and cell division, attenuate tumor growth and cancer cell survival (35). On the other hand increasing of Bax/Bcl-2 ratio up-regulates caspase-3 and releases cytochrome C from mitochondria and finally induces cell apoptosis (36). Therefore, an increased Bax/Bcl-2 ratio in breast cancer tissues of mice treated with VOE250, supports inhibition of cell survival or induction of apoptotic cascade by VOE*.* It was shown that Viola tricolor decreases cell viability of MCF-7, Neuro2a cell lines (23). However, our pervious study shown that *Viola odorata* hydro-alcoholic extract decreased cell viability of MDA-MB-468 cell line (37). Our findings present the first evidence that VOE might reduce the tumor growth via inhibitory effect on cell proliferation and induction of apoptosis in 4T1 cell line through modulation of genes involved in apoptotic signaling pathway. 

It should be noted that the rate of tumor necrosis (non-apoptotic cell death) in the experimental groups was not different compared to the control group. Meanwhile, necrotic rate showed no statistical correlation with tumor volume. Similar to the present study, *Morioka et al., *reported that tumor volume was not parallel with the necrosis values in the mouse tumor xenograft model (38), whereas others have revealed that tumor volume may increase with increasing of tumor necrosis (39).

 The results of histological examination of tissue metastasis showed that VOE in VOE250 and VOE150 group decreased liver metastasis while, lung metastasis was influenced only by VOE250. In contrast, regarding spleen metastasis there was no statistical difference between the extract-treated and control groups. Metastasis is an exceedingly complex process regulated by various factors, such as angiogenesis factor (VEGF) (40), matrix metalloproteinases (MMPs) (41), transcription factors (NF-kB, HIF-alpha ) (42, 43) and antioxidant enzymes (CAT and SOD).Virtually, all well-characterized antioxidant enzymes (CAT and SOD) have an important role in the growth and metastasis of tumors (44). Previous studies have shown that CAT decreases metastasis in liver cancer (44). SOD level in cancer patients with and without lymph node metastases is high and decreases after the operation (45). Thus, the present findings suggest that the VOE not only inhibits the tumor growth and cell proliferation of implanted 4T1 cells, but also it might decrease the lung and liver metastasis through affecting antioxidant enzyme activity and/or regulatory metastatic factors. Further researches (*Viola odorata* effect on VEGF, MMPs, NF-kB and HIF-alpha) can be helpful to clarify the *V. odorata* anti-metastasis mechanism.

Another aim of this study was to evaluate correlation between serum tumor markers (CEA and CA 15-3) and liver enzymes (GGT, ALP and LDH) levels with tumor size and metastatic rate. CEA and CA15-3 are frequently used for monitoring the response to treatment in breast cancer patients (46). Recent studies have also demonstrated that there is a significant linear correlation between CEA and CA15-3 levels with tumor size, rate of metastasis, and advanced stage in breast cancer (46).* Lumachi et al*. reported that there was no linear correlation between both CEA and CA15-3 and the other variables except for CA 15-3 vs. tumor size (47). In our study, no linear correlation was found between the CEA or CA15-3 levels with tumor size and metastatic rate. It can be mentioned that CEA and CA15-3 are not reliable for monitoring the response to treatment in 4T1 breast cancer model because of their low sensitivity and specificity(48).

GGT is an oncofetal protein, whose levels are elevated in malignant tumors( 49 ) and its antioxidant activity can modulate the proliferative/apoptotic balance in tumor progression (50). However, elevation of ALP in breast cancer patients with bone and/or liver metastasis was reported (51), Some recent studies have demonstrated that ALP enzyme activity levels show no significant difference compared to controls (45).Although, in our study metastatic rate was decreased, VOE showed no statistical inhibitory effect on GGT and ALP levels. In addition, no correlation was found between metastatic rate and the enzymes level. *Elhassaneen et al.,* denoted ALP activity increased in carbon tetrachloride (CCL_4_)-treated rats, but administration of *Viola odorata *blossom powder (0.2 to 1.6 g/100g) prevented increasing of ALP activity (3). Probably, VOE may change the level of ALP/GGT activity when used in higher dose in cancer models. 

LDH is an enzyme that catalyzes the conversion of lactate to pyruvate and increase in the serum of cancer patients (52). Our results showed that VOE decreased LDH level in VOE250 group. In VOE250 group with lower LDH level, the Ki-67 positive cancer cells were notably reduced and Bax/Bcl-2 ratio was enhanced. This results were in accordance with *Wang et al.* who found that breast cancer tumorigenicity inhibited by preventing the LDH gene expression, leading to induction of oxidative stress, apoptosis, and reduction of Ki-67 positive cancer cells (53). Therefore, decrease in serum LDH in *V. odorata* (VOE250)-treated group may be directly associated to tumor growth reduction. 

## Conclusion

In conclusion, the present study shows that extract of *V. odorata *can inhibit growth tumor and reduce the metastatic rate in the lungs and liver and also can affect antioxidant enzyme activity and apoptosis in breast cancer. Consequently, *V. odorata* extract may be considered as a potential therapeutic agent in breast cancer. Further studies are useful to clarify molecular mechanisms of *V. odorata *on treatment of breast cancer*.*
